# Myocardial Scar on Surface ECG: Selvester Score, but Not Fragmentation, Predicts Response to CRT

**DOI:** 10.1155/2020/2036545

**Published:** 2020-09-21

**Authors:** Martina Nesti, Alessandro Paoletti Perini, Rossella Bani, Stella Cartei, Luca Checchi, Giuseppe Ricciardi, Paolo Pieragnoli, Federica Michelotti, Giosuè Mascioli, Elena Cavarretta, Luigi Sciarra

**Affiliations:** ^1^Cardiovascular and Neurological Department, San Donato Hospital, Arezzo, Italy; ^2^Cardiology and Electrophysiology Unit, Santa Maria Nuova Hospital, Florence, Italy; ^3^Department of Medical, Surgical and Neuro Sciences, Diagnostic Imaging, University of Siena, Siena, Italy; ^4^Azienda Ospedaliera Universitaria Senese, Siena, Italy; ^5^Cardiology, San Giuseppe Hospital, Empoli, Florence, Italy; ^6^University of Florence, Arrhythmic Disease Unit, Firenze, Italy; ^7^Cliniche Humanitas Gavazzeni, Bergamo, Italy; ^8^Department of Medical-Surgical Sciences and Biotechnologies, Sapienza University of Rome, Latina, Italy; ^9^Mediterranea Cardiocentro, Napoli, Italy; ^10^Department of Cardiology, Policlinico Casilino, Rome, Italy

## Abstract

**Purpose:**

Myocardial scar is directly related to the response to CRT after implantation. The extent of myocardial scar can be detected not only by cardiac magnetic resonance but also by two electrocardiographic scores: fragmented QRS (fQRS) and Selvester score (SSc). The aim of our study is to compare the role of baseline SSc and fQRS in predicting response to CRT in a cohort of heart failure patients with true left bundle branch block (LBBB). As a secondary endpoint, we assessed the association of both scores with overall and cardiac mortality, heart failure hospitalizations, ventricular arrhythmias requiring ICD intervention, and major adverse cardiovascular event (MACE).

**Methods:**

We evaluated fQRS and SSc of 178 consecutive HF patients with severe systolic dysfunction (LVEF ≤ 35%), NYHA class II-III despite optimal medical treatment, and true-LBBB. Response to CRT was defined as the improvement of LVEF of at least 10% or as the reduction of LVESV of at least 15% at a 6-month follow-up. Each endpoint was related to fQRS and SSc.

**Results:**

SSc ≥7 was significantly associated with the absence of echocardiographic response to CRT (OR: 0.327; 95% C.I. 0.155–0.689; *p*=0.003), while the presence of fQRS at baseline ECG was not (OR: 1.133; 95% C.I. 0.539–2.381; *p*=0.742). No correlation was found between SSc and overall mortality, cardiac death, ventricular arrhythmias, hospitalizations due to heart failure, or for MACE. Similar results were observed between fQRS and all secondary endpoints.

**Conclusion:**

In HF patients with true-LBBB and LVEF ≤35% eligible for CRT, myocardial scar assessed by calculating the SSc on preimplant ECG is an independent predictor of nonresponse after multiple adjustments. Neither SSc nor fQRS is associated with overall and cardiac death, ventricular arrhythmias, or hospitalization for heart failure at a 24-month follow-up.

## 1. Introduction

Cardiac resynchronization therapy (CRT) is an important nonpharmacological therapy in end-stage heart failure. It is effective in improving symptoms, reversing ventricular remodeling, and reducing mortality. Although several patients meet the inclusion criteria for CRT implantation, not all of them are CRT responders [1], and patients' selection is a pivotal step in improving the overall efficacy of CRT treatment. It has been shown that the extent of myocardial scar is directly related to the response to CRT in ischemic patients [2, 3]. Detection of myocardial scar by delayed enhancement cardiac magnetic resonance (CMR) is an independent predictor of appropriate implantable cardioverter defibrillator (ICD) therapies for malignant ventricular tachyarrhythmias in CRT candidates [4]. However, CMR, probably for costs reasons, has not been widely routinely implemented in clinical practice.

However, detection of myocardial scar is possible not solely by CMR, as the presence of scars causes heterogeneous ventricular activity and alteration in the QRS morphology, leading to QRS complexes fragmentation [5], which may be observed on the 12-lead ECG, a relatively inexpensive and routine test. Previous studies reported that the presence of fragmented QRS (fQRS) on the 12-lead ECG is a sensitive and highly specific sign for myocardial scar [6] and that is also associated with clinical events in ischemic and dilated cardiomyopathies [7, 8].

Selvester et al. also showed that myocardial scar all around left ventricle (LV) produced characteristic and quantifiable changes [9] on surface ECG and developed a score, the Selvester score (SSc), strongly correlated with postmortem anatomic scar size. The first version of the score could only be used in the absence of confounders, such as left bundle branch block (LBBB). Subsequently, its criteria were modified and validated for being used also in LBBB patients (LBBB-SSc) [10].

In the present investigation, to make more accurate our results avoiding confounder data, we analyze only patients with “true-LBBB” [11], which was related to a higher event-free survival rate and a better echocardiographic response to CRT when compared to traditional LBBB [12].

The primary endpoint of the present study is to compare the role of baseline SSc and fQRS in predicting response to CRT in a cohort of HF patients with true-LBBB. As a secondary endpoint, we aimed to evaluate the association of both scores with clinical outcomes, such as overall and cardiac mortality, HF hospitalizations, incidence of ventricular arrhythmias requiring ICD intervention, and major adverse cardiovascular event (MACE).

## 2. Methods

### 2.1. Study Population

**Figure 1:**
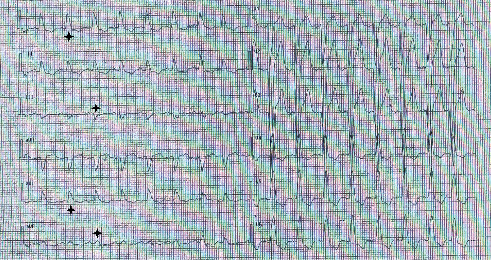
ECG showing true left bundle branch block: QRS duration 160 myocardial scar (male), QS in lead *V*_1_ and rS in lead *V*_2_, plus mid-QRS notching in all involved leads (*V*_1_ and *V*_2_; *V*_5_ and *V*_6_; DI and aVL). QRS complexes do not show fragmentation according to Das' criteria. SSc is 10.

We enrolled all consecutive HF patients with severe systolic dysfunction (LVEF ≤35%), NYHA class II-III despite optimal medical treatment and true-LBBB, who underwent CRT-D implantation from 2012 to 2016 in two Italian Centers (Azienda Ospedaliero-Universitaria Careggi, Firenze, and Cliniche Humanitas Gavazzeni, Bergamo). As previously described by Strauss et al. [11], true-LBBB was defined as the presence of QRS duration ≥140 ms (men) or ≥130 ms (women), QS or RS in leads *V*_1_ and *V*_2_, and mid-QRS notching or slurring in ≥2 of leads *V*_1_, *V*_2_, *V*_5_, *V*_6_, *I*, and aVL (Figure 1). The study is in accordance with the 1976 Declaration of Helsinki and its later amendments and was approved by the local ethics committee. Written informed consent for research purposes was obtained from all patients.

### 2.2. ICDs Programming

Detection of ventricular arrhythmias was programmed for all devices in 3 consecutive zones, with limits slightly varying per manufacturer: a monitor zone (from 150 to 185 bpm), a ventricular tachycardia (VT) zone (from 185 to 210 bpm), and a fast VT/ventricular fibrillation (VF) zone (>210 bpm). Discrimination for atrial arrhythmia was enabled. Therapy settings were adapted only when clinically indicated (e.g., hemodynamically well-tolerated VT at high rate and sustained and/or symptomatic VT in the monitor zone).

### 2.3. ECG Analysis

At both institutions, a standard 12-lead ECG (25 mm/s, 10 mm/mV) was performed before implantation with a commercially available electrocardiograph providing the automatic assessment of QRS duration and filed in a dedicated binder. For the present study, a single experienced cardiologist was blinded to all data but gender and age evaluated all ECGs. ECGs were excluded from analysis in case of poor quality, missing lead information or ventricular paced rhythm.

The SSc criteria described by Strauss and Selvester [13] (Table 1) were applied to assess the burden of MS for every patient. In this scoring method, ranging from 0 to 33, each point correlates with a 3% burden of LV scar. Finally, according to Das et al. [6], fQRS was diagnosed in the presence of various RSR' patterns with or without a *Q* wave, with >2 R waves (R'), >2 notches in the *R* wave, or >2 notches in the downstroke or upstroke of the S wave, in 2 contiguous leads.

### 2.4. Follow-Up

For all patients, a six-month follow-up included echocardiography and device interrogation. Response to CRT was defined as either the reduction of LV end-systolic volume (LVESV) of at least 15% or as the improvement of LVEF of at least 10%. All episodes of VT/VF leading to appropriate therapy were prospectively collected. Hospitalizations were assessed through hospital discharge reports. Deaths and cause of death were assessed by telephonic interviews.

### 2.5. Statistical Analysis

Continuous variables were reported as mean ± standard deviation or as median (25th–75th percentile), according to their distribution; comparisons were made by the independent samples Student's *t*-test or by the Mann–Whitney *U* test, as appropriate, while data at baseline and at follow-up were compared by the paired samples Student's *t*-test or by the related samples Wilcoxon signed rank test. Categorical variables were reported as raw data and percentages and compared by the chi-square test. Correlations were assessed by Spearman's test.

The median value of LBBB-SSc was selected as cutoff for subsequent analysis, as suggested by Wieslander et al. [10]. A bivariate logistic regression analysis was performed to detect the relationship between SSc, presence of fQRS, and response to CRT. The model included fQRS, SSc, and all variables that were significant at univariate analysis. Two different models were run, testing the SSc as either a dichotomous (greater than or equal to the median value) (model 1) or a continuous variable (model 2). Calibration of the multivariate models was assessed by the Hosmer–Lemeshow test. Cox regression analysis was performed to evaluate the incidence of clinical events according to SSc and fQRS. For all tests, a two-tailed *p* value < 0.05 was regarded as significant. Data were analyzed by means of IBM-SPSS 20.0 (SPSS Inc., Chicago, IL, USA).

## 3. Results

### 3.1. Study Population

178 consecutive heart failure patients with true-LBBB underwent CRT-D implantation in the two centers. Mean age at implantation was 70 ± 10 years, males were 120 (70%), and mean QRS duration was 161 ± 18 ms. All patients were on optimized medical therapy. Nonischemic dilated cardiomyopathy (NIDC) was the prevalent underlying disease (64%). Among ischemic patients, (64, 35.9%) and (42, 65.6%) had a previous myocardial infarction. Patients' baseline demographic, clinical, and echocardiographic characteristics are summarized in Table 2. Median SSc in the overall population was 7 (4–10): significantly higher scores were observed for ischemic patients, for whom it was 8 (5–12), while in NIDC, it was 6 (3–9) (*p* < 0.001). No significant correlations were observed between SSc and either QRS duration (Spearman's rho 0.038, *p*=0.608), age (Spearman's rho 0.028, *p*=0.714), or rhythm at implant (median value for AF 8 (6–13) and for sinus rhythm 6 (4–10), *p*=0.078).

Overall, 74 patients (41.6%) showed QRS fragmentation at their preimplant ECG: in ischemic patients, 34 had fQRS (53.1%), while in nonischemic ones, the prevalence was significantly lower (35.7%) (*p*=0.024). Fragmentation was associated with longer QRS duration (164 ± 20 ms vs 158 ± 17 ms, *p*=0.048) and with higher SSc (median 7 (5–12) vs 6 (3–9), *p*=0.004), but not with age (70 ± 10 years vs 70 ± 9 years, *p*=0.665) and rhythm at implant (*p*=0.894).

During the study, paroxysmal AF was detected in 31 patients who were in SR at implantation (19.6%); two patients developed persistent/permanent AF after the first six months of follow-up. Overall, the median value of biventricular pacing was 98% (95%–99%). No lead failures, dislodgements, or loss of capture were observed.

### 3.2. Primary Endpoint/Response to CRT

**Figure 2:**
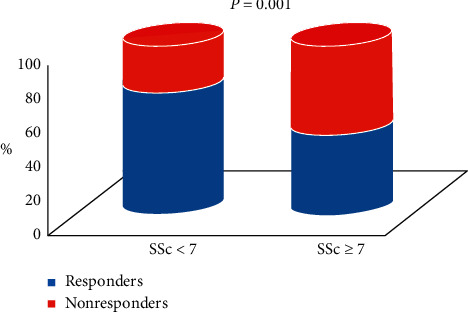
Distribution of responders and nonresponders according to the median value of the Selvester score.

**Figure 3:**
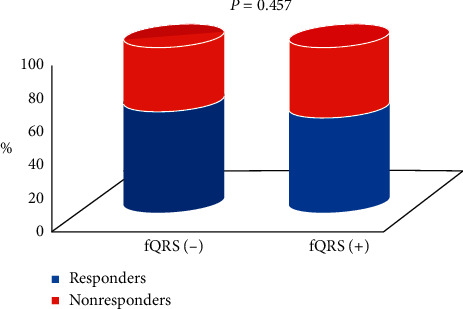
Distribution of responders and nonresponders according to the presence of fragmentation of the QRS complex.

At 6 months, echocardiographic response was observed in 106 out of 178 patients (59.5%). QRS duration was similarly reduced in both groups (Table 3). Among responders, 62 (58.5%) were male and 44 (41.5%) were female. Responders were more likely nondiabetic (80.2% vs 65.3, *p*=0.022), in sinus rhythm at time of implantation (94.3% vs 80.6%, *p*=0.004), and with lower preimplant LVEF (26.8% ± 5.5 vs 28.6% ± 5.5, *p*=0.037); SSc was significantly lower in responders than in nonresponders (5 (3–8) vs 8 (6–12), respectively, *p* < 0.001) (Figure 2), while no difference was observed for fQRS, which was present in 39.6% of responders and in 44.4% of nonresponders (*p*=0.457) (Figure 3). No other differences were detected in baseline characteristics (Table 2).

The multivariable logistic regression model confirmed that SSc ≥ 7 was significantly associated with the absence of echocardiographic response to CRT (OR: 0.327; 95% CI. 0.155–0.689; *p*=0.003), while the presence of fQRS at baseline ECG was not (OR: 1.133; 95% CI. 0.539–2.381; *p*=0.742). Moreover, occurrence of echocardiographic response was independently associated with female gender, the absence of diabetes, sinus rhythm at implantation, and lower LVEF before CRT (Table 4). The significant negative association between high SSc values and LV reverse remodeling was confirmed also when the score was entered in the multivariable model as a continuous variable (model (2) (OR: 0.884; 95% CI. 0.813–0.960; *p*=0.004) (Table 4). The Hosmer–Lemeshow test showed that both models were well calibrated (*p*=0.940 for the former and 0.695 for the latter).

### 3.3. Secondary Endpoints

No patient was lost to follow up. Over a median follow-up of 24 (9–48) months, 28 deaths occurred (15.7%), of which 18 (10.1%) were classified as cardiac deaths. Thirty-four patients (19.1%) were admitted to hospital for acute heart failure and 20 (11.2%) experienced at least one VT/VF requiring ICD intervention. Three patients experienced inappropriate ICD therapies for supraventricular tachyarrhythmias. Distribution of SSc values and of fQRS according to clinical outcomes is reported in Table 5. At univariate Cox regression analysis, no correlation was found between SSc and overall mortality (HR: 1.002, CI: 0.937–1.073, *p*=0.943), cardiac death (HR: 1.025, CI: 0.942–1.114, *p*=0.569), ventricular arrhythmias (HR: 1.038, CI: 0.964–1.118, *p*=0.321), hospitalizations due to heart failure (HR: 1.012, CI: 0.949–1.080, *p*=0.713), or for the composite endpoint of MACE (HR: 1.026 CI: 0.982–1.072, *p*=0.247).

Similar results were observed when assessing the correlation between fQRS and all secondary endpoints (overall mortality: HR: 1.722, CI: 0.772–3.840, *p*=0.184; cardiac death: HR: 1.384, CI: 0.516–3.712, *p*=0.519; VAs: HR: 1.394, CI: 0.547–3.547, *p*=0.486; and heart failure hospitalizations: HR: 1.060, CI: 0.506–2.223, *p*=0.877). No significant correlation was observed for MACE and fQRS (HR: 1.121, CI: 0.667–1.883, *p*=0.666) either.

## 4. Discussion

In the present study, we contemporarily analyzed, for the first time, the relation between both baseline SSc and QRS fragmentation and response to treatment in heart failure patients with true-LBBB eligible for CRT. Our main findings are that preimplant SSc, but not fQRS, is independently associated with LV reverse remodeling at a 6-month follow-up.

The correlation between clinical judgement and LBBB definitions varied considerably, and it is important in the perspective of LBBB being an important selection criterion for CRT. The lack of a standardized classification of LBBB may hamper consistent selection of patients [10]. The use of true-LBBB as previously defined, which is a stricter diagnostic criterion, may, in our opinion, decrease this bias.

Both SSc and fQRS are electrocardiographic tools proved effective in identifying the presence of myocardial scar [6, 10] because scar causes electrical dyssynchrony. The presence of interventricular dyssynchrony can be observed not only by the ECG, but also by vectorcardiography and, in the prediction the amount of reverse remodeling, this latter was proved to be more accurate [14, 15]. It contains three-dimensional information of electrical forces within the heart and may provide a better description of ventricular activation wavefronts; in the studies by Maass et al. [14] and van Deursen et al. [15], a larger QRS area was associated with a larger benefit of CRT.

Furthermore, previous studies have shown that a high burden and transmural extension of myocardial scar, assessed by CMR, is associated with poor response to biventricular pacing, regardless of heart failure etiology [2, 16]. However, CMR has some limitations, as it is not a bedside or widely available technique, is a highly expensive exam, and requires a specific clinical expertise. Conversely, a 12-lead ECG is not expensive, is always available for every CRT patient, and as previously proved, is useful in the detection and quantification of myocardial scar [9, 10]. Moreover, in the presence of myocardial scar, an individually tailored CRT implantation can be performed (e.g., multipolar pacing and multisite stimulation) to improve the clinical effect of resynchronization.

Previous authors have analyzed how either SSc or fQRS may predict the response to CRT [17–21]. In a previous experience, our group showed that both the extended and the simplified version of LBBB-SSc predict LV reverse remodeling in patients with true-LBBB on their preimplant ECG. However, fragmentation was not evaluated. In the present study, for the first time, we compared these two indexes in the same cohort of patients. The lack of such an investigation was recently underlined by Strauss et al. [7].

In our study, 72 out of 178 patients (40.5%) were nonresponders. While no relationship was observed between fQRS and response, SSc ≥7 (the median value) correlated directly with the absence of reverse remodeling. These findings are in agreement with Rickard et al. [18] who showed that detection of myocardial scar by the SSc can accurately predict LV reverse remodeling in patients fulfilling the classic criteria for LBBB. Also, Atwater et al. demonstrated the independent value of this score in predicting echocardiographic response in a series of 76 consecutive CRT patients [21]. The latter also limited their investigation to patients with true-LBBB ECG pattern, which is theoretically the ideal substratum for cardiac electric resynchronization, and actually offers a substantial advantage over spurious LBBB in terms of both reduced overall mortality and heart failure hospitalizations and LV functional improvement.

In the present study, we used the median value of SSc = 7 as cutoff for the analysis after Rosengarten et al. [22] who split their population according to the median value (which was 6); in their study, patients scoring ≥6 had higher mortality risk but not augmented risk of subsequent ventricular arrhythmias.

On the other hand, the role of fQRS in predicting response to CRT is not clearly defined. While in the works of Rad et al. [23] and of Rosengarten et al. [22], the presence of fQRS on baseline ECG was associated with nonresponse, our results and those reported by Bani et al. [19] showed no differences between patients with or without fragmentation. This discrepancy may be conceivably ascribed to several factors, such as the number of patients included in the studies (from a minimum of 65 to a maximum of 233), the duration and morphology of baseline QRS (classic LBBB in one study, any kind of ECG in another study, and prolonged QRS duration (≥120 ms) excluding right bundle branch block in Assadian Rad's investigation, only true-LBBB in the present study), and the prevalence of ischemic etiology (from a minimum of 36% in the present study to a maximum of 91%).

Other authors postulated that resolution of fragmentation with CRT may be the sign of effective cardiac resynchronization, which may translate in a higher degree of reverse remodeling, as demonstrated by Celikyurt et al. in a series of 67 patients with fQRS at baseline ECG [24]. However, in their experience itself up to 59% of echocardiographic responders still showed some degree of QRS fragmentation at 6-month follow-up ECG. Moreover, in this latter study, baseline ECG duration and morphology significantly differ from those in our cohort, as the inclusion criteria was classical LBBB, and mean QRS duration was 137 ± 15 ms [22].

Myocardial scar not only impairs LV reverse remodeling but also represents a substrate for ventricular arrhythmias: Nazarian et al. demonstrated the association between myocardial scar, evaluated by CMR, and the inducibility of ventricular arrhythmias by programmed stimulation in 20 patients referred for ICD implantation [25], while Fernández-Armenta et al. showed that also in CRT candidates, the presence, size, and heterogeneity of myocardial scar observed with CMR independently predict appropriate ICD therapies [4]. On the other hand, the relation between myocardial scar detected by ECG scores and incidence of ventricular arrhythmias is controversial; in a series of 98 heart failure patients with LVEF < 40% implanted with ICD after ventricular arrhythmias induction at the electrophysiological study, Hayashi et al. did not observe any correlation between fQRS and the incidence of arrhythmic events at a mean follow-up of 87 months [8]. Conversely, in a subanalysis of the SCD-HeFT trial, the absence of myocardial scar assessed by Selvester QRS-scoring resulted associated with a significant reduction in the risk of ICD shocks [7]. In the present investigation, we could not observe a significant relation between either SSc or fQRS and incidence of ventricular arrhythmias at a median follow-up of 24 months. However, a direct comparison with the aforementioned studies is not feasible because of the relevant discrepancies in the enrolled populations, as all of our patients underwent CRT application, which has the potential capability to reduce the arrhythmic burden, while CRT-D represented only 5% in Hayashi's study and was absent in Strauss's.

Finally, in our investigation, no difference in mortality was observed either in relation to either SSc or fQRS. Similarly, SSc did not differentiate patients experiencing VT/VF, sudden cardiac death (SCD), and non-SCD in the MADIT II study [26]. On the other hand, the association between mortality rates reduction and myocardial scar by SSc and fQRS was demonstrated, respectively, by Assadian Rad et al. [23] and by Hayashi et al. [8] in two small cohorts of ICD patients. However, once more, their populations are extremely different from ours, as all our patients have true-LBBB, while in the other studies, patients had different QRS morphology (LBBB was 40% in the former study [10] and 29% in the latter [7]). In a recent meta-analysis of twelve studies enrolling up to 5009 patients, Assadian Rad et al. [23] could observe a significant association between fQRS and all-cause mortality and SCD in a population of patients affected by a broad spectrum of coronary artery disease or NIDC. However, the increase of risk was limited to patients with LVEF >35%, while patients with QRS duration ≥120 ms still had higher risk of all-cause mortality but not of SCD when fragmentation was present. Our investigation is restricted to patients affected by severe LV global systolic dysfunction (LVEF ≤35%) and true-LBBB (i.e., QRS ≥140 ms for men and ≥130 ms for women), and adds further evidence to the loose association between fQRS and midterm mortality, especially in patients treated with CRT.

On the other hand, a significant association between preimplant SSc and long-term mortality after CRT has been recently demonstrated by Atwater et al. in a retrospective study of 76 patients with true-LBBB [21]; the authors showed a significant advantage in survival at a median follow-up of 47 months for patients with the lowest burden of scar (i.e., SSc < 4) in comparison to the rest. Discrepancies with our findings, despite similar enrolled populations, might be explicated by the different length of the median follow-up (24 vs 47 months), by the different sample sizes (178 vs 76 patients) and by the different kind of implanted devices; in fact, while all patients in our study received CRT-D, Atwater et al. do not report the percentage of patients who were treated with CRT-D or only with CRT-P, but just refer to CRT. Moreover, the authors do not report any information on heart failure hospitalizations either, so that no comparison is possible for such clinical outcome.

## 5. Limitations

This study have some limitations. First, ECGs made before implantation were not performed by the same operator; therefore, it is possible that slight variations in the location of electrodes might have affected the computing of the scores. Furthermore, we did not reassess the presence of fragmentation at 6 months, as we focused our interest on the evaluation of preimplant ECGs.

Third, due to the retrospective nature of the study, we were able to distinguish between patients with ischemic and nonischemic etiology of HF and, among the former, to identify those who had previous myocardial infarction, but were not able to assess the severity of the underlying coronary disease.

Finally, the present study did not include the assessment of myocardial scar by CMR. However, our study was performed to define a clinically useful score for a tailored CRT implantation, and the assessment of effective extension of myocardial scar was not our aim. A future prospective study combining a complete preimplant ECG evaluation and CMR may help to identify the best approach to detect the amount of myocardial scar, detectable by electrocardiography, which negatively affects the response to CRT.

## 6. Conclusion

Our findings indicate that in heart failure patients with true-LBBB and LVEF ≤35% eligible for CRT, myocardial scar assessed by calculating the SSc on preimplant ECG is an independent predictor of nonresponse after multiple adjustments while by fQRS is not. Therefore, SSc should be preferred to fQRS in order to identify patients who are likely not to benefit from CRT. However, neither SSc nor fQRS resulted associated with overall and cardiac death, ventricular arrhythmias, or hospitalization for heart failure at a 24-month follow-up.

## Figures and Tables

**Table 1:** LBBB Selvester score criteria.

	Lead	LBBB Selvester score criteria	Points	Maximum points
Anterosuperior wall	I	(i) R/*Q* ≤ 1.5	1	1
		(ii) R/S ≤ 1.5		
	aVL	(i) *Q* ≥ 50 ms	2	4
		(ii) *Q* ≥ 40 ms	1	
		(iii) R/S ≤ 0.5	2	
		(iv) R/*Q* ≤ 0.5		
		(v) R/S ≤ 1.0	1	
		(vi) R/*Q* ≤ 1.0		

Inferior wall	II	(i) *Q* ≥ 40 ms	2	3
		(ii) *Q* ≥ 30 ms	1	
		(iii) R/*Q* ≤ 0.5	1	
		(iv) R/S ≤ 0.5		
	aVF	(i) *Q* ≥ 50 ms	2	3
		(ii) *Q* ≥ 40 ms	1	
		(iii) R/*Q* ≤ 0.5	1	
		(iv) R/S ≤ 0.5		

Anteroseptal wall	*V*_1_	(i) Notch first 40 ms	1	3
		(ii) *R* ≥ 0.3 mV	2	
		(iii) *R* ≥ 30 ms		
		(iv) *R* ≥ 0.2 mV	1	
		(v) *R* ≥ 20 ms		
	*V*_2_	(i) Notch first 40 ms	1	3
		(ii) *R* ≥ 0.4 mV	2	
		(iii) *R* ≥ 30 ms		
		(iv) *R* ≥ 0.3 mV	1	
		(v) *R* ≥ 20 ms		

Posterolateral wall	*V*_1_	(i) S/S' ≥ 2.0	3	3
		(ii) S/S' ≥ 1.5	2	
		(iii) S/S' ≥ 1.25	1	
	*V*_2_	(i) S/S' ≥ 2.5	3	3
		(ii) S/S' ≥ 2.0	2	
		(iii) S/S' ≥ 1.5	1	

Apical segments	*I*	(i) Any Q	1	2
		(ii) *R* ≤ 0.2 mV		
		(iii) R/*Q* ≤ 1.0	1	
		(iv) R/S ≤ 1.0		
	*V*_5_	(i) Any Q	1	4
		(ii) R/R' ≥ 2.0	2 1	
		(iii) R/R' ≥ 1.0		
		(iv) R/S ≤ 2.0		
		(v) *R* ≤ 0.5 mV	1	
	*V*_6_	(i) *Q* ≥ 20 ms	1	4
		(ii) R/R' ≥ 2.0	2 1	383
		(iii) R/R' ≥ 1.0		
		(iv) R/S ≤ 2.0		
		(v) *R* ≤ 0.6 mV	1	384

**Table 2:** Population baseline characteristics and distribution between responders and nonresponders.

Variables	All patients (*n* = 178, 100%)	Responders (*n* = 106, 59.5%)	Nonresponders(*n* = 72, 40.5%)	*p* value
Age	70 ± 10	70.4 ± 10	70 ± 9	0.729
Males	120 (67.4%)	62 (58.5)%	58 (80.6%)	0.002
Females	58 (32.6%)	44 (41.5%)	14 (19.4%)	
NYHA class II	32 (18.0%)	23 (21.7%)	9 (13.5%)	0.117
III	146 (82.0%)	83 (78.3%)	63 (87.5%)	
Ischemic etiology	64 (35.9%)	36 (34.0%)	28 (38.9%)	0.457
QRS (ms)	161 ± 18	163 ± 18	158 ± 18	0.115
AF	20 (11.2%)	6 (5.7%)	14 (19.4%)	0.004
Hypertension	85 (47.7%)	67 (63.2%)	45 (62.5%)	0.996
DM	46 (25.8%)	21 (19.8%)	25 (34.7%)	0.022
Dyslipidemia	72 (40.4%)	41 (38.7%)	31 (43.1%)	0.776
ACE-is/ARBs	160 (89.9%)	97 (91.5%)	62 (86.1%)	0.367
Beta-blockers	142 (79.8%)	89 (84.0%)	53 (73.6%)	0.127
Statins	70 (39.3%)	39 (36.8%)	31 (43.1%)	0.360
MRAs	81 (45.5%)	50 (47.2%)	30 (41.7%)	0.519
Diuretics	148 (83.1%)	86 (81.1%)	61 (84.7%)	0.354
LVEF (%)	27.5 ± 5.6	26.8 ± 5.5	28.6 ± 5.5	0.037
LVEDV (mL) ^*∗*^	192 (152–228)	193 (165–229)	177 (132–227)	0.156
LVESV (mL) ^*∗*^	137 (110–170)	140 (116–175)	135 (94–165)	0.244
LVEDD (mm)	69.2 ± 10.2	68.3 ± 9.4	70.7 ± 11.3	0.238
LVESD (mm)	57.8 ± 11.3	57.4 ± 10.8	58.5 ± 12.3	0.638
Selvester score	7 (4–10)	5 (3–8)	8 (6–12)	<0.001
Selvester score ≥ 7		42 (39.6)	47 (65.3%)	0.001
Fragmented QRS	74 (41.6%)	42 (39.6%)	32 (44.4%)	0.457

Continuous variables are expressed as mean ± SD, and compared by the independent samples Student's t-test, or as median and 25°/75° percentiles, and compared by the Mann–Whitney *U* test, as appropriate ( ^*∗*^); categorical variables as number of patients (%) and compared by the *χ*2 test. AF, atrial fibrillation; NYHA, New York Heart Association; LVEF, left ventricular ejection fraction; DM, diabetes mellitus; ACE-I, angiotensin converting enzyme-inhibitors; ARBs, angiotensin receptor blockers; MRAs, mineralocorticoid receptor antagonists; LVEDD, left ventricular end-diastolic diameter; LVESD, left ventricular end-systolic diameter; LVEDV, left ventricular end-diastolic volume; and LVESV, left ventricular end-systolic volume.

**Table 3:** Differences in echocardiographic variables and QRS duration between baseline and the 6-month follow-up and between responders and nonresponders.

Variables	Responders, baseline	Responders, 6 months	*p* value vs baseline	Nonresponders, baseline	Nonresponders, 6 months	*p* value vs baseline
LVEDD (mm)	68.3 ± 9.4	63.1 ± 11.9	0.005	70.7 ± 11.3^‡^	67.9 ± 8.3 ^§^	0.089
LVESD (mm)	57.4 ± 10.8	49.3 ± 13.5	0.001	58.5 ± 12.3^‡^	55.1 ± 9.7 ^†^	0.330
LVEDV (mL) ^*∗*^	193 (165–229)	140 (105–165)	<0.001	177 (132–227) ^‡^	184 (146–240)^!!^	0.014
LVESV (mL) ^*∗*^	140 (116–175)	83 (56–108)	<0.001	135 (94–165) ^‡^	132 (90–166)^!!^	0.243
LVEF (%)	26.8 ± 5.5	41.8 ± 9.3	<0.001	28.6 ± 5.5^§^	30.5 ± 6.6^!!^	0.018
QRS (ms)	163 ± 18	137 ± 28	<0.001	158 ± 18 ^‡^	141 ± 33^‡^	<0.001

Continuous variables are expressed as mean ± SD, and compared by Student's *t*-test for paired samples (baseline vs 6 months) and for independent samples (responder vs nonresponders), or as median and 25°/75° percentiles ( ^*∗*^), and compared by the related samples Wilcoxon signed rank test (baseline vs 6 months) and by the independent samples Mann–Whitney *U* test, as appropriate. LVEDD, left ventricular end-diastolic diameter; LVESD, left ventricular end-systolic diameter; LVEDV, left ventricular end-diastolic volume; LVESV, left ventricular end-systolic volume; and LVEF, left ventricular ejection fraction; and †Paoletti Perini is the family name.  ^‡^*p* n.s. versus responders;  ^§^*p* < 0.05 versus responders;  ^†^*p* < 0.01 versus responders; ^!!^*p* < 0.001 versus responders.

**Table 4:** Selvester score and fragmented QRS univariable and multivariable analysis for the primary endpoint (response to CRT).

	Univariable binary logistic regression			Multivariable ^*∗*^		
	O.R.	95% C.I.	*pvalue*	O.R.	95% C.I.	*pvalue*
Age	1.005	0.975–1.037	0.727			
**Gender (female)**	**2.889**	**1.434–5.823**	**0.003**	**5.027**	**2.001–12.627**	**0.001**
NYHA class	0.798	0.472–1.348	0.398			
**LVEF**	**0.943**	**0.891–0.997**	**0.039**	**0.864**	**0.801–0.933**	**<0.001**
QRS (ms)	1.014	0.997–1.031	0.117			
**AF**	**0.242**	**0.088–0.666**	**0.006**	**0.199**	**0.064–0.621**	**0.005**
Etiology (ischemic)	0.790	0.424–1.473	0.458			
Hypertension	0.998	0.485–2.053	0.996			
**Diabetes mellitus**	**0.455**	**0.230–0.899**	**0.023**	**0.331**	**0.143–0.765**	**0.010**
Dyslipidemia	0.802	0.432–1.491	0.486			
ACE-is/ARBs	1.565	0.589–4.158	0.369			
Beta-blockers	1.778	0.844–3.345	0.130			
Statins	0.751	0.407–1.386	0.360			
MRAs	1.220	0.666–2.237	0.520			
Diuretics	0.668	0.283–1.575	0.357			
**Selvester score**	**0.869**	**0.809–0.934**	**<0.001**	^*∗∗*^		
**Selvester score ≥7**	**0.335**	**0.179–0.627**	**0.001**	**0.327**	**0.155–0.689**	**0.003**
Fragmented QRS	0.793	0.431–1.461	0.457	**1.133**	**0.539–2.381**	**0.742**
LVEDD (mm)	0.976	0.939–1.016	0.976			
LVESD (mm)	0.991	0.955–1.028	0.635			
LVEDV (mL)	1.005	0.999–1.012	0.127			
LVESV (mL)	1.006	0.998–1.014	0.134			

^*∗*^Model 1 includes Selvester score ≥7.  ^*∗∗*^In model 2, Selvester score is expressed as continuous variable; OR 0.884 (0.813–0.960); *p*: 0.004. Gender (female), LVEF, AF, diabetes mellitus, and fragmented QRS showed the same OR and *p* values as in model 1. AF, atrial fibrillation; NYHA, New York Heart Association; LVEF, left ventricular ejection fraction; ACE-I, angiotensin converting enzyme-inhibitors; ARBs, angiotensin receptor blockers; MRAs, mineralocorticoid receptor antagonists; LVEDD, left ventricular end-diastolic diameter; LVESD, left ventricular end-systolic diameter; LVEDV, left ventricular end-diastolic volume; and LVESV, left ventricular end-systolic volume.

**Table 5:** Distribution of SSc values, and prevalence of fQRS, according to the occurrence of secondary endpoints.

	SSc values			Presence of fQRS (74 pts)		
	Median	25/75 percentile	*p* value	Yes	No	*p* value
VT/VF (20 pts)	7.5	4–13	0.260	10 (50%)	10 (50%)	0.417
No VT/VF (158 pts)	6	4–10		64 (40.5%)	94 (59.5%)	

HF hospitalization (34 pts)	6.5	4–13	0.432	15 (44.1%)	19 (55.9%)	0.738
No HF hospitalization (144)	6.5	4–10		59 (41%)	85 (59%)	

Cardiac death (18 pts)	7.5	5–11	0.145	15 (53.6%)	13 (46.4%)	0.161
No cardiac death (160 pts)	6	4–10		59 (36.9%)	91 (56.9%)	

All-cause death (28 pts)	7	4–11	0.377	9 (50%)	9 (50%)	0.444
No death (150 pts)	6	4–10		65 (40.6%)	95 (59.4%)	

MACE (66 pts)	7	4–12	0.065	30 (45.4%)	36 (45.6%)	0.420
No MACE (112 pts)	6	3–9		44 (39.3%)	68 (60.7%)	

Continuous variables are expressed as median and 25°/75° percentiles, and compared by the Mann–Whitney *U* test; categorical variables as number of patients (%) and compared by the *χ*2 test. SSc, Selvester score; fQRS, fragmented QRS; VT, ventricular tachycardia; VF, ventricular fibrillation; HF, heart failure; and MACE, major adverse cardiovascular event.

## Data Availability

The clinical data used to support the findings of this study are included within the article.
